# Breast Ductal Carcinoma In Situ

**DOI:** 10.1155/2012/753267

**Published:** 2012-11-06

**Authors:** Virgilio Sacchini, Lucio Fortunato, Hiram S. Cody III, Kimberly J. Van Zee, Bruno Cutuli, Bernardo Bonanni

**Affiliations:** ^1^Breast Service, Department of Surgery, Memorial Sloan-Kettering Cancer Center, 300 East 66th Street New York, NY 10065, USA; ^2^Senology Unit, Department of Surgery, San Giovanni Addolorata Hospital, 00184 Rome, Italy; ^3^Radiation Oncology Department, Polyclinique de Courlancy, 51100 Reims, France; ^4^Division of Cancer Prevention and Genetics, European Institute of Oncology, 20141 Milan, Italy

Ductal carcinoma in situ (DCIS) of the breast is becoming one of the most important diseases diagnosed in preventive medicine screening. The current age-adjusted incidence rate of DCIS is 32.5 per 100,000 women. For women 50–64 years of age, the incidence is approximately 88 per 100,000. Currently, for every 4 diagnoses of invasive breast cancer, there is 1 diagnosis of DCIS. Risk of DCIS is rare in women younger than 30 years of age and is low in women under 40 years of age, but increases steadily from ages 40–50. The risk of DCIS increases much more slowly after the age of 50, and it plateaus after the age of 60.

Assuming constant incidence and survival rates, it is estimated that more than 1 million women will be living with diagnosed DCIS by 2020, with obvious social and political health ramifications. The question scientists are facing now, and the question women are beginning to ask their breast surgeons, is whether we are overdiagnosing and overtreating this disease. 

Should we be less aggressive and more tolerant toward this disease? We are aware that DCIS is a spectrum of different diseases and that we may have overdiagnosed and overtreated to a point, but we have also likely undertreated others. Despite randomized clinical trials and evidence-based recommendations, there are important regional and geographic differences in clinical management, reflecting both a cultural bias regarding, and the heterogeneity of, this disease. 

In this current issue, the authors present a detailed overview of the state of the art of the diagnosis and treatment of this disease as well as an overview of future tendencies and research.

M. Badruddoja extensively reviews the most recent knowledge of epidemiology and risk factors for DCIS.

L. Fortunato et al. and M. Amichetti et al. review all the randomized clinical trials on DCIS, giving an unbiased interpretation of the data that is helpful in the daily management of DCIS patients.

K. Lambert et al., in their literature review facilitated by the Medline, PubMed, Embase, and Cochrane databases, consider randomized, nonrandomized, prospective, and retrospective studies with the goal of increasing understanding of the most important predictive factors for local recurrence, and of better selection of the use of radiation therapy and tamoxifen.

The still debatable issue of sentinel node biopsy in DCIS is addressed by D. Boler et al., who present their experiences and discuss literature evidence.

G. Scripcaru and I. M. Zardawi investigate the occurrence of each architectural growth pattern in mammary DCIS. Correlating the architecture with nuclear grade, they postulate that the comedo pattern can ultimately occur in all types of DCIS and therefore should not be regarded as a separate DCIS type.

 D. Coradini et al., in an elegant experiment, investigate the expression patterns of a selected panel of genes associated with cell polarity and the apical junction complex. They are able to confirm that atypical ductal hyperplasia and DCIS are part of a tumorigenic, multistep process with possible chemoprevention implications.

E. Bastiaannet et al., analyzing 8421 patients with DCIS, found no excess mortality irrespective of treatment in women older than 50 years of age, with the important clinical implication that local relapse in these women may not impact prognosis.

K. Van Zee and D. Choi et al. from Memorial Sloan-Kettering Cancer Center investigate many aspects of the biology, diagnosis, and treatment of DCIS over a 20-year period with the final development of a nomogram that incorporates many factors simultaneously to estimate the risk of local relapse in DCIS treated with conservation therapy. 

Several recent patterns-of-care studies have identified substantial variation in surgeon decision making regarding the optimal management of DCIS [1–5]. In September 2009, the National Institutes of Health convened a conference to discuss the diagnosis and management of patients with DCIS because of the complexity and discrepancy in its management [6]. 

The papers presented here represent important contributions toward a better understanding of this disease and its treatment, and emphasize the need for a multidisciplinary approach to DCIS.

*Virgilio Sacchini* *Virgilio Sacchini* *Lucio Fortunato* *Lucio Fortunato* *Hiram S. Cody III* *Hiram S. Cody III* *Kimberly J. Van Zee* *Kimberly J. Van Zee* *Bruno Cutuli* *Bruno Cutuli* *Bernardo Bonanni* *Bernardo Bonanni*
